# Retrieval of Raindrop Size Distribution Using Dual-Polarized Microwave Signals from LEO Satellites: A Feasibility Study through Simulations

**DOI:** 10.3390/s21196389

**Published:** 2021-09-24

**Authors:** Xi Shen, Defeng David Huang

**Affiliations:** Department of Electrical, Electronic and Computer Engineering, The University of Western Australia, Perth 6009, Australia; david.huang@uwa.edu.au

**Keywords:** raindrop size distribution, rain attenuation, low Earth orbit satellites, microwave propagation, polarization

## Abstract

In this paper, a novel approach for raindrop size distribution retrieval using dual-polarized microwave signals from low Earth orbit satellites is proposed. The feasibility of this approach is studied through modelling and simulating the retrieval system which includes multiple ground receivers equipped with signal-to-noise ratio estimators and a low Earth orbit satellite communicating with the receivers using both vertically and horizontally polarized signals. Our analysis suggests that the dual-polarized links offer the opportunity to estimate two independent raindrop size distribution parameters. To achieve that, the vertical and horizontal polarization attenuations need to be measured at low elevation angles where the difference between them is more distinct. Two synthetic rain fields are generated to test the performance of the retrieval. Simulation results suggest that the specific attenuations for both link types can be retrieved through a least-squares algorithm. They also confirm that the specific attenuation ratio of vertically to horizontally polarized signals can be used to retrieve the slope and intercept parameters of raindrop size distribution.

## 1. Introduction

Using the attenuation of electromagnetic signals to measure rainfall has received much attention recently. This approach is strongly supported by the theoretical basis for calculating attenuation due to the water content along a signal path, which has been established and validated by numerous propagation tests [1,2]. In 1991, it was suggested that custom-designed microwave links could be used for the reconstruction of rainfall fields [3]. The usage of the backhaul links of commercial wireless communication networks (CWCNs) for rainfall monitoring was first proposed by Messer et al. in 2006 [4] and then by Leijnse et al. in 2007 [5]. Since then, this technique has been improved rapidly through extensive studies (e.g., [6,7,8,9]). The Earth–space links of commercial geostationary Earth orbit (GEO) satellites have also been studied to serve the same purpose. It has been demonstrated that the GEO system is capable of path-integrated rain rate estimation [10,11,12], and that the estimation has a high accuracy for heavy rain events [13].

The recent development in large constellations of low Earth orbit (LEO) satellites for global broadband services hints at a new opportunity for rainfall measurements. Several companies are pursuing large constellations of LEO satellites to provide global broadband access, e.g., Starlink’s system with more than 10,000 satellites and Telesat’s system with 300 satellites [14]. Once the satellite broadband service is available to the public, it can be foreseen that millions of user terminals (ground receivers) will be deployed across the globe. A dynamic network of satellite-to-ground links will be formed on an unprecedented scale. It will become a very large sensor network that could provide off-the-shelf signal attenuation data for the observations of the atmosphere. Meanwhile, because of the increasing demand for multimedia applications and thus the requirement of high data rates, satellites broadband services need to use Ku (12~18 GHz), Ka (26.5~40 GHz), and/or Q/V (40~50 GHz) frequency bands [15]. Signals at these frequencies are severely attenuated by gas, cloud and rain in the atmosphere [16]. They are especially sensitive to liquid water, which indicates that measuring precipitation through them can be a valid approach. The opportunistic use of satellite communication links can provide global real-time observations at a very low cost.

One of the key advantages of using a LEO satellite system for rainfall estimation is that it can offer three-dimensional (3-D) rain field tomography. First proposed by Huang et al. in 2016 [17], this approach exploits the similarity in the revolutionary motion between the LEO satellites and the signal source in computed tomography (CT) [18] and performs tomographic reconstruction of rain attenuation fields. Various investigations have been carried out recently to further examine this approach. For example, it has been suggested that 3-D attenuation fields can be retrieved by using the estimated signal-to-noise ratio (SNR) at the ground receivers and an iterative method to estimate the sky noise [19]. Furthermore, some signal processing strategies have been explored to optimize the performance of the rain field retrieval [20,21,22].

The attenuation of microwave links also provides the opportunity for raindrop size distribution (DSD) measurements. Knowledge of the DSD is fundamental to quantitative precipitation estimation and realistic DSD representation is very important to atmospheric research. Studies have suggested that the DSD is best modeled by a gamma distribution [23], which is in the following form:

(1) $$N\left(D\right)=N_{0}D^{\mu }e^{-\Lambda D},$$

in which D is the equivolumetric raindrop diameter in mm, $N_{0}$ is the intercept parameter in mm^−1^⋅m^−3^, μ is a dimensionless shape parameter and Λ is the slope parameter in mm^−1^. One straightforward approach to estimate the three parameters is through three different attenuation measurements. For instance, previous studies have shown that attenuation measurements of multi-frequency links can be used to retrieve the parameters [24,25]. Taking advantage of the oblateness of large raindrops, the approach of using attenuation measured by multi-polarization links has also been investigated [26,27]. Furthermore, the multi-polarization and multi-frequency approaches can be combined together to improve the performance of the DSD retrieval [28]. On the other hand, it has been suggested that not all three parameters are always needed for the representation of DSD. Some studies use fixed μ models (e.g., [25,29]), while others identify a μ−Λ relationship (e.g., [30,31]) so that the number of independent parameters is also reduced to two. Both approaches have their merits and other factors such as rain types and locations determine which approach performs better.

To date, existing studies use ground links at fixed locations to retrieve the path-averaged DSD parameters. In this paper, we will explore the possibility of exploiting the tomographic capability of moving signal links offered by LEO satellites. Realistic system models are proposed for the satellite communication system in which both vertically and horizontally polarized links are in operation. Synthetic DSD fields are adopted to suit the purpose of the study, and we use the Pruppacher–Beard raindrop shape model [32] and the T-matrix method [33] to calculate the attenuation caused by electromagnetic scattering. Our calculation shows that the elevation angle of the link does not affect the specific attenuation very much for horizontally polarized signals. While for vertical polarization, the increase in specific attenuation for the same rain field could be more than 10% when the elevation angle changes from 40 to 90 degrees. The two attenuation fields retrieved by the two differently polarized links are examined for the purpose of DSD estimation. It is found that a linear relationship can be identified between the slope parameter and the retrieved specific attenuation ratio of vertically to horizontally polarized signals. Using this relationship, we examine a fixed μ model and investigate the system’s ability to retrieve the slope (Λ) and intercept ($N_{0})$ parameters. Simulation results show that for areas of high specific attenuations, the retrieval is more accurate, thereby both the retrieved slope and intercept parameters have a close agreement with their true values.

The main contributions of this study are summarized as follows.

- A theoretical model is built for the novel approach for raindrop size distribution retrieval using dual-polarized microwave signals from LEO satellites;
- The feasibility of the approach is investigated through simulations of synthetic rain events and realistic satellite communication systems;
- It is confirmed that the specific attenuation ratio of vertically to horizontally polarized signals can be used to retrieve the slope and intercept parameters of DSD.

The rest of the paper is organized as follows. Section 2 describes the satellite signal model, and explains how the SNR at the receivers is related to the path-integrated attenuation. It also establishes the DSD parametric model and examines the theoretical basis of DSD retrieval. In Section 3, simulation results of the synthetic DSD fields, the retrieved attenuation fields and DSD parameters are presented and discussed. Section 4 concludes the paper.

## 2. Model and Methods

### 2.1. Satellite Signal Model

We use the same satellite signal model presented in previous studies on rain field [19] and cloud field retrieval [34]. The model consists of a LEO satellite passing directly over the area of interest and multiple ground receivers equipped with electronically steerable antennas. It should be noted that the receivers designed for satellite broadband will have a minimum elevation angle requirement in order to satisfy the minimum signal quality requirement for communication. When the minimum elevation angle is met, the satellite-to-ground link becomes operational and the SNRs at the ground receivers are estimated for the purpose of measuring the path-integrated attenuation. For one overpass, suppose that k denotes the elevation angle in degrees, and ρ(k) is the SNR at the receivers in the form of [19,20]

(2) $$\rho \left(k\right)=C\left(k\right)-F_{n}\left(k\right)-A_{I}\left(k\right)-A_{F}\left(k\right),$$

where $A_{I}\left(k\right)$ and $A_{F}\left(k\right)\;$are the path-integrated rain attenuation and the free space path loss, in decibels, respectively. Parameter C(k) is an unknown baseline value for a receiver, which is mainly determined by the power of the transmitted signal, the antenna gain and the noise temperature of the receiving system. Noise figure $F_{n}\left(k\right)$ is a function of the sky temperature and the noise temperature of the receiving system. We assume that the noise temperature of the receiving system is known so $F_{n}\left(k\right)$ is a function of the unknown sky noise. A detailed description of C(k), $A_{F}\left(k\right)$ and $F_{n}\left(k\right)$ can be found in [19] (pp. 5437–5438).

It is assumed that each receiver is operating on a dual-polarization mode, i.e., both vertical and horizontal polarizations are used at the same time. Hence, the system offers two estimated SNRs, namely ${\hat{\rho }}_{V}\left(k\right)$ and ${\hat{\rho }}_{H}\left(k\right)$ at every receiver, for measuring path-integrated attenuations.

### 2.2. Specific Attenuation and DSD

**Figure 1:**
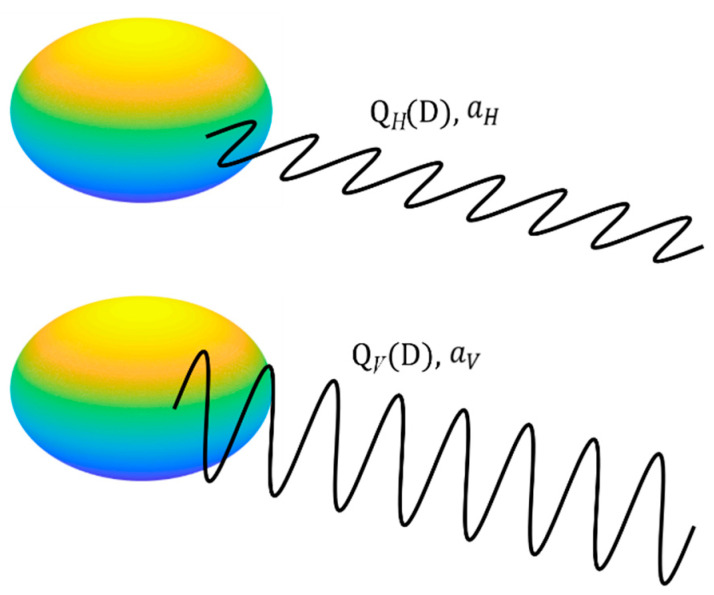
An oblate raindrop has different extinction cross sections and causes different attenuations for vertically and horizontally polarized signals.

The relationship between the specific attenuation (a in dB/km) and the DSD of (1) is given by [35]

(3) $$a=4.343\times \int_{0}^{\infty }N\left(D\right)Q\left(D\right)dD,$$

where Q(D) is the extinction cross section of the raindrop (in m^2^), which is determined by the raindrop shape, temperature, and frequency, direction and polarization of the microwave signal. Because large raindrops are in an oblate shape, their extinction cross sections will be different for vertically and horizontally polarized microwave signals with the same wavelength. Figure 1 shows a sketch of the raindrop and signals with different polarizations, where $Q_{H}\left(D\right)$ and $Q_{V}\left(D\right)$ are the extinction cross sections for horizontally and vertically polarized links, respectively. As a result, the specific attenuations in the rain field, namely $a_{V}$ and $a_{H}$, will also be different.

The exact shape of a raindrop is usually complicated as it is determined by the surface tension of the water and the air flow around it as it falls. However, there are simplified models available to suit the requirement of calculating Q(D). In this paper, we adopt the widely used Pruppacher–Beard model [32] to characterize the raindrop shape. The axial ratio of the raindrop is given by

(4) $$\frac{y}{x}=1.03-0.062D,$$

where x is the semininor axis and y is the semimajor axis of the raindrop. This model yields a very good approximation of raindrop shapes according to their diameters up to 7 mm [36], which enables us to utilize the T-matrix method [33,37] for calculating their extinction cross sections. The T-matrix method proposed by Waterman [38] is a powerful tool for accurately computing electromagnetic scattering by nonspherical particles. It is based on directly solving Maxwell’s equations and yet its efficiency enables very fast computation. The fact that raindrops are approximately spheroids further simplifies the T-matrix calculation.

### 2.3. Retrieval Method

The retrieval method aims to estimate the DSD parameters by using the attenuation measurements of both vertically and horizontally polarized signal links. For the LEO satellite communication systems, the elevation angle is constantly changing during an overpass. Assuming the raindrops have zero canting angles, we examine the extinction cross section for various drop sizes for 30 GHz signals at different elevation angles. Table 1 lists the values of $Q_{H}\left(D\right)$ when the elevation angle is at 40, 55, 70 and 90 degrees for raindrop sizes from 0.5 to 6.0 mm in diameter. Table 2 lists the $Q_{V}\left(D\right)$ to $Q_{H}\left(D\right)$ ratio to show the difference between the two. As the elevation angle goes higher and as the drop size becomes smaller, the gap between $Q_{V}\left(D\right)$ and $Q_{H}\left(D\right)$ diminishes. This indicates that for better measurement of the DSD, we need to examine the horizontal and vertical polarization attenuations at lower elevation angles where their difference is more distinct.

With SNRs at multiple ground receivers estimated for both horizontally and vertically polarized links elevated at 40° (${\hat{\rho }}_{H}\left(40\right)$ and ${\hat{\rho }}_{V}\left(40\right)$), the specific attenuations $a_{H}\left(40\right)$ and $a_{V}\left(40\right)$ for a targeted area can be retrieved through a tomographic algorithm. This retrieval process involves dividing the area of interest into voxels, solving the specific attenuations of each voxel using algorithms such as least-squares [39] and iteratively updating the noise figures of the sky noises (see [19] (p. 5439) for details).

With the two specific attenuations ready, from Equations (1) and (3), we obtain

(5) $$\frac{a_{V}}{a_{H}}=\frac{\int_{0}^{\infty }D^{\mu }e^{-\Lambda D}Q_{V}\left(D\right)dD}{\int_{0}^{\infty }D^{\mu }e^{-\Lambda D}Q_{H}\left(D\right)dD}\;,$$

in which parameter $N_{0}$ is eliminated. By assuming that the shape parameter μ is a fixed known value, Equation (5) provides a one-to-one mapping between $a_{V}/a_{H}$ and Λ. When Λ is successfully estimated, $N_{0}$ can then be solved using Equations (1) and (3).

## 3. Simulations and Results

Our proposed retrieval method was examined through a series of simulations. We use synthetic DSD fields combined with a realistic LEO satellite communication system to simulate the estimated SNRs. The retrieval results of the specific attenuations and the DSD parameters are compared with their true values.

### 3.1. Synthetic DSD Field

**Figure 2:**
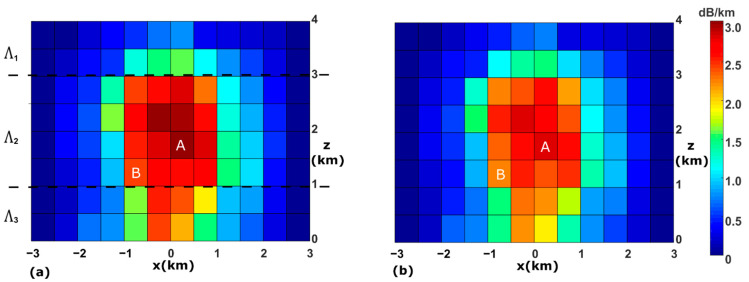
(**a**) The true specific attenuation field for horizontally polarized signals with 40° elevation angle. Warmer colors indicate higher specific attenuations. (**b**) The true specific attenuation field for vertically polarized signals with 40° elevation angle.

A vertical DSD field is generated synthetically for our simulations. The field is considered to remain static during the overpass time of the LEO satellite (approximately 289 s in our simulations). It is 6 km in length and 4 km in height, and divided into 12×8=96 voxels, with each voxels measuring 500 m by 500 m. The DSD profile for each voxel is then generated as follows. Firstly, the specific attenuation for each voxel for horizontally polarized 30 GHz signals with 40° elevation angle ($a_{H}\left(40\right)$) is generated according to a localized frontal rainfall event [19] simulated by the Weather Research and Forecasting (WRF) model. This event is a subset of a larger convection-permitting simulation covering the southern part of the Great Barrier Reef and adjacent coastline. As shown in Figure 2a, the entire rain field is contained within the 96 voxels. The specific attenuation varies from voxel to voxel. For instance, voxel A has the highest specific attenuation ($a_{H}\left(40\right)$ = 3.23 dB/km) and some voxels on the edge of the field have zero dB/km specific attenuations. Secondly, the shape parameter μ for all voxels is assumed to be 1, which is in line with the WDM6 Scheme of WRF. Thirdly, the field is divided into three layers (middle layer 2 km in height, top and bottom layers 1 km in height) and the slope parameters ($\Lambda_{1}$, $\Lambda_{2}$ and $\Lambda_{3}$) are assigned for each layer (see Figure 2a). This is based on the assumption that for a particular rain event, the slope parameter is more spatially homogeneous than the intercept parameter [40]. According to the probability density function of Λ in recent studies [41], $\Lambda_{1}$ and $\Lambda_{3}$ are set to be 2.9 mm^−1^ and 4.1 mm^−1^ for the bottom and top layer, respectively. Initially assigned to 3.5 mm^−1^, $\Lambda_{2}$ for the middle layer is able to take different values in the following simulations so that we can test the performance of the DSD retrieval. Finally, the intercept parameter $N_{0}$ for each voxel is calculated from $a_{H}\left(40\right)$ and Λ using Equations (1) and (3). The canting angles of raindrops follow a Gaussian distribution with a mean of 0° and a standard deviation of 2° [42,43]. In the calculation a MATLAB implementation of the T-Matrix method [44] is applied and the integral is computed numerically from D_min_ = 0.01 mm to D_max_ = 7.0 mm in 0.01 mm increments. Simulation results suggest that $N_{0}$ for voxels with nonzero specific attenuations is between $6.75\times 10^{2}$ and $5.46\times 10^{4}$ mm^−1^⋅m^−3^ when $\Lambda_{2}$ is 3.5 mm^−1^. With $N_{0}\;$ and Λ ready, the specific attenuation for any elevation angle and for either polarizations can then be calculated. Figure 2b shows the true field of $a_{V}\left(40\right)$ for comparison, in which the specific attenuation for voxel A is 2.99 dB/km, 7.4% less than $a_{H}\left(40\right)$.

### 3.2. Retrieval of Specific Attenuation

In the simulations, we use a LEO satellite with a circular Keplerian motion trajectory of an orbital height of 1000 km and an inclination angle of 96°. The trajectory is on the same vertical plane as the rain field. There are 14 receivers evenly placed at the ground level, from −3.25 km to 3.25 km, with any two adjacent receivers being 0.5 km apart. The SNRs for the horizontally and vertically polarized links are generated using exactly the same parametric configurations as in previous studies [19,20]. It is worth noting that the estimation error of the SNRs is taken into account by using the Cramer–Rao lower bound (CRLB) [45]. This introduces an error of approximately 0.01 dB in the SNR measurements ${\hat{\rho }}_{V}\left(k\right)$ and ${\hat{\rho }}_{H}\left(k\right)$. We assume that there are 50 SNR measurements from 50 different elevation angles available, evenly distributed in the overpass of the satellite.

**Figure 3:**
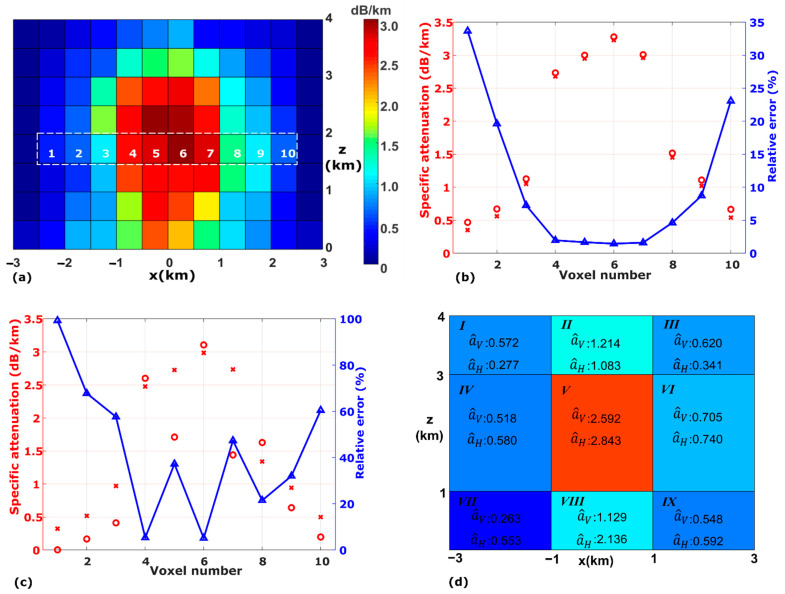
(**a**) The retrieved specific attenuation field for horizontally polarized signals at 40 degrees. (**b**) Retrieval results of horizontal specific attenuations for the ten voxels in the 4th row from the bottom in 100 simulations: the true specific attenuations are marked by red crosses and the average retrieved specific attenuations are marked by red circles. The blue triangles show the relative error. (**c**) Retrieval results of vertical-specific attenuation for the ten voxels in the 4th row from the bottom in 100 simulations: the true specific attenuations are marked by red crosses and the average retrieved specific attenuations are marked by red circles. The blue triangles show the relative error. (**d**) The newly divided nine areas and the retrieved specific attenuations for both horizontal and vertical polarizations.

We use a differential approach [20] and an iterative process [19] to estimate the specific attenuations of all voxels as well as the noise figure $F_{n}$. Ideally, we want to retrieve ${\hat{a}}_{H}\left(40\right)$ and ${\hat{a}}_{V}\left(40\right)$ only from ${\hat{\rho }}_{H}\left(40\right)$ and ${\hat{\rho }}_{V}\left(40\right)$. However, results suggest that links at 40° only are not sufficient for the least-squares algorithm to solve the unknowns. On the other hand, for horizontally polarized links, the change in attenuation caused by the change in the elevation angle is relatively small within the targeted range of Λ. Table 3 lists the $a_{H}\left(k\right)$ to $a_{V}\left(40\right)$ ratio for different elevation angles and different values of Λ calculated by the T-Matrix method. It shows that the change in specific attenuation as the elevation angle increases never exceeds approximately 1%. In other words, considering $a_{H}$ being unchanged for all elevation angles will not introduce a significant error in the retrieval algorithm. Consequently, we just utilize the measurements of ${\hat{\rho }}_{H}$ from all elevation angles to estimate ${\hat{a}}_{H}$, which will be regarded as ${\hat{a}}_{H}\left(40\right)$. Same as previous studies [19], we use an iterative process (summarized in Algorithm 1) to update the noise figure for a more accurate estimation of ${\hat{a}}_{H}\left(40\right)$. Figure 3a shows the estimated ${\hat{a}}_{H}\left(40\right)$ for the entire field in one simulation. It can be seen that the retrieved field shows very close agreement with the true field in Figure 2a. In 100 independent simulations, we record ${\hat{a}}_{H}\left(40\right)$ for the 10 voxels in the 4th row from the bottom (marked in Figure 3a) with nonzero true specific attenuations. As shown in Figure 3b, the true specific attenuations are marked by the red crosses and the average retrieved specific attenuations over 100 simulations are marked by the red circles. The average relative error, which is calculated by averaging over 100 simulations the ratio of the absolute error to the true specific attenuation, is shown by the blue triangles. It can be concluded that retrieval of specific attenuations is relatively more accurate for voxels with high specific attenuations. More specifically, the relative error for the middle four voxels is below 2%.
**Algorithm 1.** The iterative process used to update the noise figure. **Input**:$\;{\hat{\rho }}_{H}\left(k\right)$, k=1,2, …, M; **Output**: $\alpha_{H}$ for all voxels initialize $F_{n}=0$; **while** not the last iteration **do**  Use least-squares to solve $\alpha_{H}$,  Calculate $A_{I}\left(k\right)$ using $\alpha_{H}$, Update$\;F_{n}$ using$\;\;\alpha_{H}$ and$\;\;A_{I}$.

For vertically polarized links, the change in $a_{V}$ with the elevation angle will be too large to ignore. Table 4 shows the value of $a_{V}\left(k\right)/a_{V}\left(40\right)$ for different values of Λ. It can be seen that $a_{V}\left(54.34\right)$ already exceeds $a_{V}\left(40\right)$ by over 4% when Λ is 2.8 mm^−1^. As a result, only ${\hat{\rho }}_{V}\left(k\right)$ measured close to 40° can be used in the least-squares algorithm without introducing too much error. It should also be noted that the noise figure is now already calculated based on ${\hat{a}}_{H}\left(40\right)$ so the least-squares algorithm can be directly applied to estimate ${\hat{a}}_{V}\left(40\right)$ without engaging the iterative process of Algorithm 1. Although this would again introduce some error, simulations suggest that the error can be considered negligible. Multiple simulations suggest that the retrieved field is erroneous due to underdetermination in the least-squares algorithm when less than eight SNR measurements from 40° (${\hat{\rho }}_{V}\left(40\right)$ to ${\hat{\rho }}_{V}\left(51.2\right))$ are used. Figure 3c shows the retrieval of ${\hat{a}}_{V}\left(40\right)$ for the same 10 voxels as in Figure 3b. Here, eight SNR measurements are used but the relative errors for individual voxels are much higher than in the retrieval of $a_{H}$. For instance, for voxel 5, the relative error of estimated ${\hat{a}}_{V}\left(40\right)\;$is 39%. This indicates that using estimated ${\hat{a}}_{V}\left(40\right)/{\hat{a}}_{H}\left(40\right)$ of individual voxels to calculate the slope parameter Λ will lead to very large errors. As a result, we propose a new model for retrieving ${\hat{a}}_{V}\left(40\right)$ and thus ${\hat{a}}_{V}\left(40\right)/{\hat{a}}_{H}\left(40\right)$, taking advantage of the assumption that Λ does not change within a layer.

Before retriving ${\hat{a}}_{V}\left(40\right)$, the entire field is divided into three layers according to Λ and then nine areas as shown in Figure 3d, with each of area being 2 km in width. The same least-squares algorithm is applied to retrieve the specific attenuation for vertical polarization for each area. Figure 3d shows the retrieved ${\hat{a}}_{V}\left(40\right)$ field in one simulation, in which SNR measurements at eight different elevation angles (${\hat{\rho }}_{V}\left(40\right)$ to ${\hat{\rho }}_{V}\left(51.2\right)$) are used. For each area, ${\hat{a}}_{H}\left(40\right)$ is then calculated by averaging the previously retrieved ${\hat{a}}_{H}\left(40\right)$ over all of the voxels within the area. For example, ${\hat{a}}_{H}\left(40\right)$ for area *I* is the average of eight voxels in the top left in Figure 3a and for area *V* it is the average of 16 voxels in the center. The calculated ${\hat{a}}_{H}\left(40\right)$ for each of the areas is also shown in Figure 3d. Note that the relative errors in ${\hat{a}}_{H}\left(40\right)$ for the center 16 voxels are very low so ${\hat{a}}_{H}\left(40\right)$ for area *V* is expected to be very accurate. Hence, using ${\hat{a}}_{V}\left(40\right)/{\hat{a}}_{H}\left(40\right)$ of area *V* is the best choice for estimating the slope parameter $\Lambda_{2}.$

**Figure 4:**
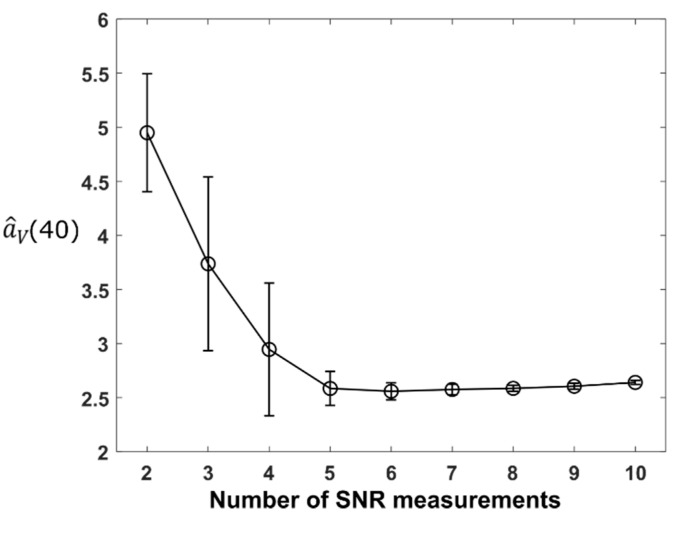
Using different numbers of SNR measurements to retrieve the specific attenuation for vertically polarized link at 40 degrees. The circles mark the average retrieved specific attenuations for area V across 100 independent simulations and the error bars show the standard deviation.

In order to achieve the best result of solving ${\hat{a}}_{V}\left(40\right)$, we need to use only SNR measurements close to a 40° elevation angle but also to ensure there are enough data points for the least-squares algorithm to work. Nine groups of simulations were carried out to find the optimal balance between the two requirements. In each group, a certain number of SNR measurements from 40° onwards are used to retrieve ${\hat{a}}_{V}\left(40\right)$, i.e., two measurements (${\hat{\rho }}_{V}\left(40\right)$ and$\;{\hat{\rho }}_{V}\left(41.4\right))$ are used for the first group and sequentially ten measurements (${\hat{\rho }}_{V}\left(40\right)$ to ${\hat{\rho }}_{V}\left(54.3\right))$ for the ninth group. Each group contains 100 independent simulations. Figure 4 shows the mean and standard deviation of ${\hat{a}}_{V}\left(40\right)$ for area *V* in the 100 simulations for each group. It can be seen that the standard deviation is very large when less than five SNR measurements are used, indicating that the retrieved ${\hat{a}}_{V}\left(40\right)$ could be erroneous. These simulations suggest that using 7 to 10 SNR measurements will lead to the best overall results for retrieved ${\hat{a}}_{V}\left(40\right)$.

### 3.3. Retrieval of DSD Parameters

**Figure 5:**
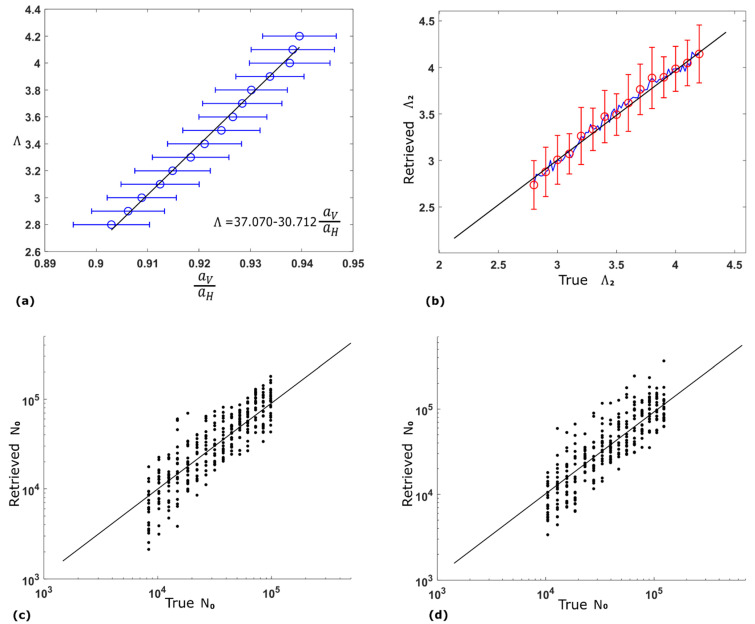
(**a**) The retrieved specific attenuation ratio when the slope parameter for area *V* takes different values. Each blue circle represents the mean for 100 simulations, and each error bar shows the standard deviation. The black line is the linear regression result over the blue circles, which is also given by the equation. (**b**) The retrieved slope parameter using the linear relationship. The blue line shows the average for 100 simulations and the red bars show the standard deviation at certain values. (**c**) Scatter plot of the retrieved intercept parameter for voxel A. (**d**) Scatter plot of the retrieved intercept parameter for voxel B.

As the ratio of $a_{V}\left(40\right)/a_{H}\left(40\right)$ holds the key to retrieving the slope parameter Λ, we examine the retrieved ratio for different slope parameters. Because the retrieval of specific attenuations is relatively more accurate for areas with higher values, we will focus on area *V* in Figure 3d and evaluate the performance of retrieving $\Lambda_{2}$. In total 15 simulation groups are designed, in each of which parameter $\Lambda_{2}$ takes values from 2.8 mm^−1^ to 4.2 mm^−1^ in 0.1 mm^−1^ increments. In each of the simulation groups, 100 independent simulations are carried out. Figure 5a shows the retrieved ${\hat{a}}_{V}\left(40\right)/{\hat{a}}_{H}\left(40\right)$ for area *V*, in which the blue circles mark the average retrieved ratio across 100 simulations and the error bars show the standard deviation. It is found that the relationship between true $\Lambda_{2}$ and the mean of the retrieved ${\hat{a}}_{V}\left(40\right)/{\hat{a}}_{H}\left(40\right)$ is almost linear. Using a linear least-squares regression fit of the blue circles, this linear relationship is generated and shown by the black line and the equation in the figure.

Our following simulations utilize the linear relationship above to infer $\Lambda_{2}$ from the retrieved ${\hat{a}}_{V}\left(40\right)/{\hat{a}}_{H}\left(40\right)$. In the new 141 groups of 100 independent simulations, the true parameter $\Lambda_{2}$ takes values from 2.8 mm^−1^ to 4.2 mm^−1^ in 0.01 mm^−1^ increments, and the retrieved $\Lambda_{2}$ is calculated from the retrieved ${\hat{a}}_{V}\left(40\right)/{\hat{a}}_{H}\left(40\right)$ using the linear equation in Figure 5a. Figure 5b shows the retrieval outcome, in which the blue line is the average retrieved $\Lambda_{2}$ and the black line is a reference line with a gradient of 1. The red bars show the standard deviation across 100 simulations when the true $\Lambda_{2}$ is at some selected values.

Furthermore, the intercept parameter $N_{0}$ for each of the 16 voxels in area *V* of Figure 3d is calculated from the retrieved $a_{H}\left(40\right)$ and $\Lambda_{2}$, and then compared with its true value. In Figure 5c, the retrieved $N_{0}$ is compared with its true value for voxel A in Figure 2a in 15 different $\Lambda_{2}$ (true $\Lambda_{2}$ being from 2.8 mm^−1^ to 4.2 mm^−1^ in 0.1 mm^−1^ increments) with each one containing 20 independent simulations. The black line is also a reference line with a gradient of 1. The same was carried out for voxel B in Figure 2a where the true $N_{0}$ is less than that for voxel A, and the results are shown in Figure 5d. It can be concluded from the two figures that the retrieved $N_{0}$ has a close agreement with the true$\;N_{0}$ for the 15 different $\Lambda_{2}$. More simulations confirm that the retrieval outcomes are very similar for all of the 16 voxels in area *V*. As the retrieved ${\hat{a}}_{H}\left(40\right)$ is quite accurate, the level of error in retrieved $N_{0}$ is mainly due to the fluctuation in retrieved $\Lambda_{2}$, which can be seen in Figure 5b.

### 3.4. Simulations of Another Rain Event

**Figure 6:**
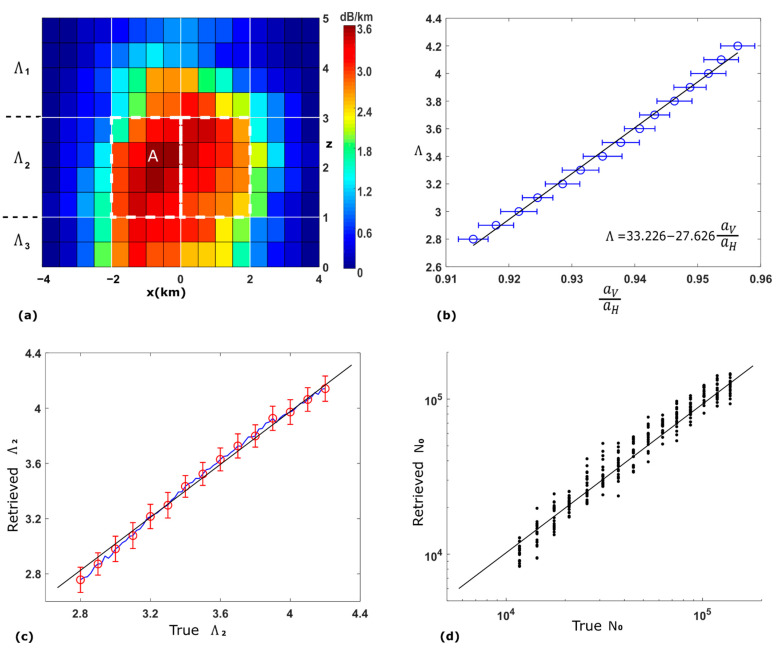
(**a**) Second rain event: the true specific attenuation field for horizontally polarized signals with 40° elevation angle. (**b**) The retrieved specific attenuation ratio for different slope parameter values. Each blue circle represents the mean for 100 simulations, and each error bar shows the standard deviation. The black line is the linear regression result over the blue circles, which is also given by the equation. (**c**) The retrieved slope parameter using the linear relationship. The blue line shows the average for 100 simulations and the red bars show the standard deviation at certain values. (**d**) Scatter plot of the retrieved intercept parameter for voxel A.

A second rain event is utilized to further verify the performance of the proposed method. Shown in Figure 6a, it is an idealized convective storm generated by WRF using the same resolution settings as before. The vertical attenuation field measures 8 km (width) by 5 km (height). The highest specific attenuation (for horizontal polarization at 40°) in all voxels is larger than the frontal rain field in Figure 2a. The DSD profile for each voxel is generated following the exact same process. The entire field is redivided into 4×3 (marked by white solid lines in Figure 6a) areas, two of which (marked by white dashed lines in Figure 6a) are used to retrieve the DSD parameters. Figure 6b shows the retrieved ${\hat{a}}_{V}\left(40\right)/{\hat{a}}_{H}\left(40\right)$ when $\Lambda_{2}$ takes different values, in which the blue circles mark the average retrieved ratio across 100 simulations and the error bars show the standard deviation. Again, using a linear least-squares regression fit of the blue circles, a linear relationship is identified and shown by the black line and the equation in Figure 6b. Figure 6c shows the retrieved $\Lambda_{2}$ using the identified linear relationship, in which the blue line is the average retrieved $\Lambda_{2}$ and the black line is a reference line with a gradient of 1. The red bars show the standard deviation across 100 simulations when the true $\Lambda_{2}$ is at the same selected values as in Figure 5b. Figure 6d is the scatter plot of the retrieved $N_{0}$ for voxel A in Figure 6a.

The results above suggest that the performance of retrieval is consistent for both the synthetic convective storm and the simulated frontal rainfall. The proposed methods display great potential in retrieving the slope and intercept parameters of DSD. Although only the retrieval of vertical DSD fields is discussed in this paper, it can be inferred that 3-D retrieval can be achieved through the aggregation of a series of retrieved vertical fields.

## 4. Conclusions

In this paper, we use computer simulations to explore the feasibility of using dual-polarized microwave signals from LEO satellites to measure the raindrop size distribution. Our analysis suggests that measurements of attenuations on both horizontally and vertically polarized microwave links offer the opportunity to estimate two independent DSD parameters. As the difference between the two specific attenuations is crucial for retrieving the slope parameter and becomes less distinct as the elevation angle of the link goes higher, its retrieval needs to be performed at low elevation angles, preferably close to the minimum elevation angle of the receivers. Simulation results show that using SNR measurements from all elevation angles to retrieve the specific attenuation for horizontally polarized signals through a least-squares algorithm is feasible. Particularly for voxels with heavy rain, the retrieval error is less than 2%. It is also confirmed that the specific attenuation for vertically polarized signals can be retrieved using a few SNR measurements taken close to the minimum elevation angle. In order to achieve the precision needed for estimating the DSD parameters, the retrieval algorithm is performed on a large area that covers several voxels. It is suggested that the specific attenuation ratio of vertically to horizontally polarized signals can be used through a linear relationship to estimate the slope parameter of the DSD, and that the intercept parameter can also be retrieved using the estimated slope parameter. The retrieved values for both parameters closely agree with their true values.

As the estimation of DSD parameters demands high accuracy in the specific attenuation measurements, the following sources of error in the system need to be further examined in future work. The first source of error comes from the definition of the voxels, which inevitably introduces a difference from the true field. This difference can be reduced by using a finer resolution grid, but it also means that the retrieval task will become more difficult. The second source of error is the unknown sky noise. The attenuation field is initially retrieved by assuming the sky noise is zero. Although the iterative process can update the sky noise and fine-tune the retrieval result, the final error is still significant. The third is the SNR estimation error, which can be reduced by designing good estimators but cannot be completely eliminated. We expect that the proposed approach can provide even better DSD estimations if these sources of error can be further mitigated.

## Figures and Tables

**Table 1:** The extinction cross section for horizontal polarization calculated by the T-Matrix method for various raindrop sizes and elevation angles.

$Q_{H}\left(D\right)$ $\left(\times 10^{-8}\;m^{2}\right)$		Elevation Angle (°)			
		40	55	70	90
*D* (mm)	0.5	1.1337	1.1336	1.1335	1.1335
	1.0	23.634	23.499	23.389	23.327
	2.0	509.52	507.46	505.76	504.82
	4.0	4019.0	4139.8	4243.7	4302.9
	6.0	9064.2	9318.2	9487.6	9562.0

**Table 2:** The extinction cross section ratio of vertical to horizontal polarizations calculated by the T-Matrix method for various raindrop sizes and elevation angles.

$Q_{V}\left(D\right)/Q_{H}\left(D\right)$		Elevation Angle (°)			
		40	55	70	90
*D* (mm)	0.5	0.9989	0.9994	0.9998	1
	1.0	0.9700	0.9831	0.9939	1
	2.0	0.9045	0.9461	0.9807	1
	4.0	0.8585	0.9220	0.9727	1
	6.0	0.8366	0.9191	0.9746	1

**Table 3:** The specific attenuation for a horizontally polarized link changes while the elevation angle increases from 40 degrees. Nonetheless, the maximum change is only about 1%.

$a_{H}\left(k\right)/$ $a_{H}\left(40\right)$		$\Lambda ({\mathrm{mm}}^{-1})$			
		2.8	3.3	3.8	4.2
k (°)	42.80	1.0000	0.9996	0.9992	0.9991
	54.34	1.0000	0.9977	0.9961	0.9955
	66.68	1.0001	0.9959	0.9933	0.9921
	88.72	1.0003	0.9943	0.9909	0.9893

**Table 4:** The specific attenuation for a vertically polarized link changes while the elevation angle increases from 40 degrees. The change is much more significant compared to that for a horizontally polarized link.

$a_{V}\left(k\right)/$ $a_{V}\left(40\right)$		$\Lambda ({\mathrm{mm}}^{-1})$			
		2.8	3.3	3.8	4.2
k (°)	42.80	1.0090	1.0064	1.0052	1.0044
	54.34	1.0436	1.0344	1.0267	1.0216
	66.68	1.0773	1.0600	1.0467	1.0381
	88.72	1.1057	1.0819	1.0639	1.0523
